# Trim-Away in adult animals through Nano-ERASER and its application in cancer therapy

**DOI:** 10.21203/rs.3.rs-2298306/v1

**Published:** 2023-01-17

**Authors:** Mingming Wang, Junfeng Wang, Yuzhen Wang, Manikanda Keerthi Raja, Gourab Gupta, Xiangxiang Hu, Shanshan Shi, Hexin Chen, Daping Fan, Peisheng Xu

**Affiliations:** 1 Department of Discovery and Biomedical Sciences, College of Pharmacy, University of South Carolina, 715 Sumter, Columbia, SC 29208 (USA); 2 Department of Cell Biology and Anatomy, School of Medicine, University of South Carolina, 6439 Garners Ferry Rod, Columbia, SC 29209 (USA); 3 Department of Biological Sciences, University of South Carolina, *715 Sumter, Columbia, SC 29208 (USA)*

**Keywords:** Trim-Away, adult animal, Nano-ERASER, PD-L1, antibody, cancer

## Abstract

Trim-Away is a versatile intracellular protein degradation pathway that has been extensively explored *in vitro*. However, the *in vivo* application of Trim-Away is limited at oocyte and zygote stages due to the lack of an *in vivo* practical approach for intracellular antibody delivery. To broaden the application of Trim-Away, especially for clinical use, we developed a nanogel-based Nano-ERASER system. Here, we demonstrated that the intracellular delivery of anti-programmed cell death ligand 1 (PD-L1) antibody through Nano-ERASER could effectively deplete PD-L1 in triple negative breast cancer (TNBC) cells and induce cancer cell death. Furthermore, with the help of a tumor tissue-targeted nanogel, anti-PD-L1 antibody-loaded Nano-ERASER effectively inhibited tumor progression in a TNBC mouse model. These results confirmed that Nano-ERASER realized Trim-Away in adult animals for the first time, which could be an effective tool for disease treatment and studying gene/protein function both *in vitro* and *in vivo*.

## Introduction

Trim-Away is a recently discovered endogenous protein degradation mechanism that can selectively degrade proteins in mammalian cells by intracellularly delivering antibodies.^1^ It utilizes an intrinsic cellular self-defense machinery, which involves the intracellular supply of an antibody, binding of the antibody to its target protein, formation of tripartite motif containing-21 (TRIM21) antibody/target protein complex and ubiquitination, and proteasome-mediated degradation of the complex. Attributed to its merit in highly selective and rapidly degrading intracellular protein, A broad spectrum of proteins have been successfully down-regulated in various cells with Trim-Away.^1–7^

Despite being broadly explored *in vitro*, the *in vivo* application of Trim-Away is limited in oocyte and zygote stages due to the lack of a practical approach to delivering antibodies in animal models, not to mention in humans for treating diseases.^2–6, 8–11^ By far, the intracellular delivery of antibodies in most Trim-Away applications was realized through micro-injection and electroporation, which are impractical for an *in vivo* setting. Given the challenge and the potential benefit of targeting undruggable targets, there is an urgent need for a clinical translational approach to enable Trim-Away-based protein degradation for *in vivo* application.

Our group recently developed a Nano-ERASER technology, which realizes Trim-Away in cells without microinjection or electroporation through intracellular delivery and tracelessly releasing antibodies.^12^ Nano-ERASER is an antibody-conjugated polymer nanogel (NG) system that can effectively intracellularly deliver and release antibodies and induce the degradation of a specific endogenous protein. The potential therapeutic efficacy of Nano-ERASER has been validated by depleting COPZ1 protein and causing the death of cancer cells.^12^

Anti-PD-L1 antibody, which interrupts the binding of PD-L1 on cancer cells and/or myeloid cells to PD-1 on T cells, and thereby restores the cytotoxicity of effector T cells, has been clinically used for the treatment of various types of cancers through a cancer immunotherapy mechanism.^13–18^ However, for patients with solid tumors, including triple negative breast cancer (TNBC), only 15–25% respond to anti-PD-L1 Abs.^17, 18^ New evidence shows that PD-L1 exists in various forms, including cell membranous, secreted, and nuclear states. In addition to interacting with PD-1 on the T cell surface, PD-L1 exerts tumor-promoting effects in various ways.^19, 20^ First, PD-L1 may promote cancer cell growth through intracellular pro-survival signaling independent of PD-1.^21^ Second, PD-L1 can be translocated into the nucleus and acts on multiple targets to improve DNA stability and cancer cell repair, thereby inhibiting apoptosis and promoting cancer growth.^22^ Data showed that PD-L1 knockout in cancer cells significantly inhibited tumor growth,^23, 24^ suggesting the importance of the PD-L1/PD-1 interaction-independent tumor-promoting role of cancer cell PD-L1. Furthermore, a recent study revealed that intracellular PD-L1 plays a vital role in promoting metastasis and modulating epithelial-to-mesenchymal transition (EMT) in TNBC.^25, 26^

**Fig.1:**
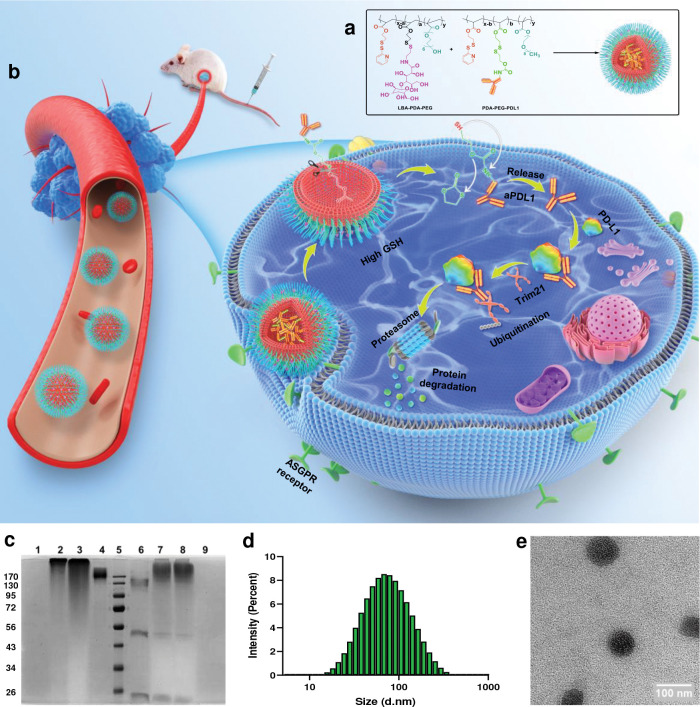
The synthesis and characterization of TN-PDL1 for in vivo Trim-Away. **a,** The scheme for the fabrication of TN-PDL1. **b,** The proposed *in vivo* Trim-Away pathway for TN-PDL1. **c,** PAGE gel electrophoresis image. Lanes 1 and 9 were PDA-PEG polymer, lane 2 and 8 were N-PDL1 nanogel, lane 3 and 7 were aPDL1-polymer conjugates, lane 4 and 6 were free aPDL1, lane 5 was protein maker. Samples in lane 6–9 were incubated in 10 mM GSH at 37 °C for 1 hr. **d,** The size distribution of TN-PDL1. **e,** TEM image of TN-PDL1. Scale bar in **e** was 100 nm.

To extend the application of Trim-Away for *in vivo* application and cancer therapy, we designed a tumor tissue-targeted Nano-ERASER system, TN-PDL1, to deliver anti-PD-L1 antibody (aPDL1) selectively into cancer cells and assessed its effect on tumor progression in a TNBC mouse model (Fig. 1a–b). It was revealed that TN-PDL1 could effectively reduce the expression of PD-L1 in cancer cells both *in vitro* and *in vivo*. In addition, the degradation of PD-L1 induced the death of the cancer cells and prevented the progression of the tumor. Here, we demonstrated for the first time that Trim-Away can be realized in adult animals and exhibits a therapeutic effect in a disease animal model.

## RESULTS

### Generation of an aPDL1-loaded tumor-targeted nanogel (TN-PDL1)

**Fig. 2:**
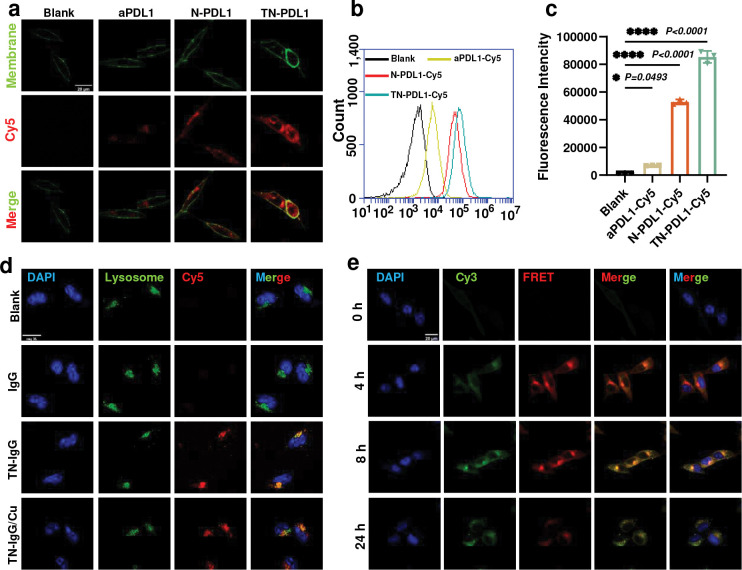
Cellular uptake and lysosomal escape of TN-PDL1. **a,** Representative confocal images of 4T1 cells incubated with Cy5-labeled aPDL1, N-PDL1, and TN-PDL1 for 3 h. The membrane of the cells was stained using CellBrite Steady membrane staining kits. **b,** The spectra of flow cytometry analysis for the uptake of Cy5-labeled aPDL1, N-PDL1, and TN-PDL1. **c,** Quantitative analysis of Cy5-labeled aPDL1, N-PDL1, and TN-PDL1 entering 4T1 cells. n=3. Mean ± SD, ****P < 0.0001. **d,** Representative confocal images of 4T1 cells incubated with Cy5-labeled IgG, TN-IgG, and TN-IgG supplemented with 10 μM CuCl_2_ for 3 h. The lysosomes were stained with LysoTracker Green. The cell nuclei were stained with DAPI. **e.** The FRET signal of TN-IgG-Cy3-Cy5 detected by confocal microscopy. The FRET signals were collected with the excitation of 555 nm, and emission of Cy3 (560–600 nm) and Cy5 (640–700 nm). The scale bars were 20 μm.

To enable Nano-ERASER based Trim-Away for cancer therapy, we generated TN-PDL1 by functionalizing Nano-ERASER with an ASGPR receptor targeting ligand, lactobionic acid (LBA) (Supplementary Scheme 1).^27^ TN-PDL1 was fabricated following our published protocol except replacing the previous antibody and targeting ligand with aPDL1 and LBA through amide linkage (Fig. 1a),^12^ respectively. Agarose gel electrophoresis confirmed that aPDL1 could be encapsulated into the nanogel and the encapsulated aPDL1 can be successfully released under a reducing environment mimicking intracellular conditions (Fig. 1c). Dynamic light scattering revealed that the size distribution of the nanogel was 78.6 ± 35.6 nm (PDI: 0.2, Fig. 1d); transmission electron microscope found that the nanogel had a spherical shape (Fig. 1e). Zeta sizer shown that TN-PDL1 had a surface charge of −18±1.19 mV (Supplementary Fig. 1a). To evaluate the targeting effect of the LBA ligand for TNBC, Cy5 labeled-aPDL1 was adopted. Nanogels with LBA modification (TN-PDL1) and without LBA modification (N-PDL1) were incubated with 4T1 cells. After three hours of co-culture, the cellular uptake of the nanogels was observed by confocal microscope and quantified by flow cytometry. Fig. 2a–c and Supplementary Fig. 1b showed that the functionalization of LBA greatly improved the cellular uptake of TN-PDL1, evidenced by the boosted red signals inside the cells. Similar results were also confirmed by flow cytometry (Fig. 2b–c). As expected, only a weak red signal was observed in the free aPDL1 treated cells, suggesting the necessity of an effective tool to transfer aPDL1 into the cell. Fig. 2c revealed that there was a greater than seven-fold increase in fluorescent intensity in TN-PDL1 treated cells than that of free aPDL1 treated cells.

### Subcellular localization and lysosomal escaping of TN-PDL1

To study the lysosomal escaping of an antibody inside the cells, Cy5 labeled IgG (IgG-Cy5) was employed, due to its inert effect, as a model antibody to yield TN-IgG-Cy5 nanogel. It was reported that copper ions facilitate the lysosomal escaping of chelators.^28^ 4T1 cells were incubated with TN-IgG-Cy5 for 3 h with or without the addition of 10 μM CuCl_2_, and observed with confocal microscopy. The abundant red signals inside TN-IgG-Cy5 treated cells proved that targeted NGs could effectively enter the cells (Fig. 2d). It was noticed that most red signals in the TN-IgG-Cy5 treated cells overlapped with the green signals of Lysotraker, indicating the slow escaping of the TN-IgG from the lysosomes. In contrast, the addition of copper ions facilitated the lysosomal escaping of TN-IgG, evidenced by more standing alone red spots in the TN-IgG-Cy5/Cu treated cells (Fig. 2d). Since copper ions has been reported for promoting lysosomal escaping,^28^ we attribute the quick lysosomal escaping of the NGs to the positive charges of the copper ions.

### Intracellular release of antibody from nanogel

To monitor the intracellular release of antibody, which is a prerequisite of Trim-Away, Förster resonance energy transfer (FRET) technology was employed. Due to its inert bioactive property, control IgG was selected as a model antibody. Cy5-labeled IgG and Cy3-labeled PDA-PEG polymer were used to yield Cy3/Cy5 dual-labeled NGs. After 4 h of incubation with TN-IgG-Cy3-Cy5, the cells were excited with a 555 nm laser. Strong Cy5 (red, emission 640–700 nm) signals accompanied by weak Cy3 (green, emission 560–600 nm) signals were observed in the 4T1 cells (Fig. 2e), indicating an apparent FRET phenomenon due to the closely associated polymer (Cy3) and antibody (Cy5). At 8 h post-incubation, the increased Cy3 signals and declined Cy5 signals were observed, indicating a partial liberation of Cy5-labelled antibody from the NGs. The subsequent fading of red signals at 24 h suggests the almost full release of antibodies inside the cell. These results suggest targeted nanogels (TN) can effectively deliver an antibody into cells and release it in a timed manner.

### TN-PDL1 depletes both total PD-L1 and membrane PD-L1 in 4T1 cells

**Fig. 3:**
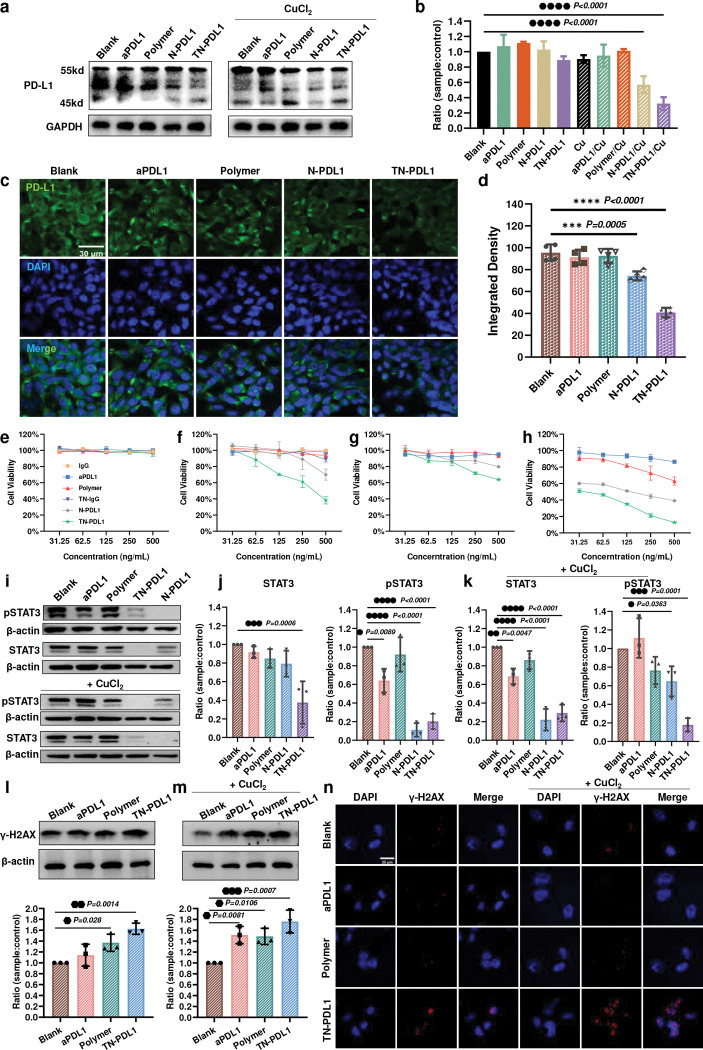
TN-PDL1 depletes PD-L1, inhibits the proliferation of 4T1 cells, prevents the activation of STAT3, and inhibits DNA damage repair. **a,** Protein expression level of PD-L1 in 4T1 cells after receiving aPDL1 (0.1 μg/mL), N-PDL1, and TN-PDL1 treatment with or without CuCl_2_ (10 μM) supplement for 16 h. **b,** Quantitative analysis of PD-L1 expression in **a**. **c,** The representative immunofluorescence images of the membrane PD-L1 expression after being treated with aPDL1 (0.05 μg/mL), N-PDL1, and TN-PDL1 for 24 h. **d,** Quantitative analysis of PD-L1 intensity in **c**. **e-h,** The cell killing effect of TN-DPL1. MTT assays were used to quantify the viability of 4T1 cells after being treated with different concentrations of IgG, aPDL1, polymer, TN-IgG, N-PDL1, and TN-PDL1 for 24 h with (**f**, **h**) or without (**e**, **g**) 10 μM CuCl_2_ and the addition of PBMCs (**g**, **h**) (ratio of PBMCs to 4T1 cells is 10:1). Statistical analysis was performed via ANOVA one-way test, n=3, Mean ± SD. **i,** Protein expression level of STAT3 and pSTAT3 in 4T1 cells after receiving aPDL1, polymer, N-PDL1, and TN-PDL1 treatment with or without the supplement of CuCl_2_ (10 μM) for 16 h at an aPDL1 equivalent dose of 0.25 μg/mL. **j-k**, Quantitative analysis of STAT3 and pSTAT3 expression in **i**. Protein expression of γ-H2AX in 4T1 cells after receiving aPDL1, polymer, and TN-PDL1 treatment with (**m**) or without (**l**) 10 μM CuCl_2_ supplement. The dose of the treatments were at an aPDL1 equivalent dose of 0.1 μg/mL. Statistical analysis was performed via ANOVA one-way test, n=3, Mean ± SD. **n,** Representative confocal images of γ-H2AX expression in 4T1 cells after being incubated with aPDL1 (0.05 μg/mL), polymer, and TN-PDL1 for 24 h with or without 10 μM CuCl_2_ supplement. The cell nuclei were stained with DAPI. The scale bar was 20 μm.

For the success of Trim-Away, the availability of intracellular TRIM21 is required. Supplementary Fig. 3 revealed that 4T1 cells express a high level of TRIM21, which satisfies the prerequisite of Trim-Away. Since TN-IgG can effectively carry IgG into the cells and release IgG intracellularly (Fig. 2d), we first probed whether TN-IgG could induce the degradation of PD-L1. Supplementary Fig. 2 found that the intracellular delivery of IgG did not affect the expression of PD-L1. However, TN-PDL1 effectively reduced PD-L1 level in 4T1 cells at the dose of 100 ng/ml after 6 h of treatment, suggesting the quick action of Trim-Away (Supplementary Fig. 3a–b). Furthermore, TN-PDL1 exhibited higher efficacy in degrading PD-L1 than N-PDL1, attributed to its enhanced cellular uptake. There was no significant difference between the treatments with or without the addition of CuCl_2_ (Supplementary Fig. 3a–b). Interestingly, after 24 h of treatment, N-PDL1 and TN-PDL1 coupled with CuCl_2_ induced more than 40% and 60% PD-L1 reduction (Fig. 3a–b), respectively. In contrast, TN-PDL1 alone did not cause significant PD-L1 change in the same period, suggesting insufficient TN-PDL1 was released inside the cytoplasm due to its relatively poor lysosomal escaping capacity (Fig. 2d). By combining the results in Supplementary Fig. 2 and Fig. 3a–b, we can conclude that the reduction of PD-L1 in TN-PDL1 treated cells was due to the specific degradation effect of TN-PDL1, not the carrier system.

To further probe the effect of TN-PDL1 on the expression of membrane PD-L1, immunocytochemistry was employed. Fig. 3c–d revealed that the degradation of intracellular PD-L1 also resulted in reduced PD-L1 expression on the cell membrane of 4T1 cells. This suggests that TN-PDL1 may exhibit immunotherapeutic effect by preventing the binding of PD-1 on T cells to the PD-L1 on the TNBC cells.

### TN-PDL1 inhibits the proliferation of cancer cells

The cell proliferation inhibitory effect of TN-PDL1 on 4T1 cells was evaluated by MTT assay. Since our previous study found that the combination of copper ions and polymer-carriers kills cancer cells,^29^ polymer control at the corresponding dose of its nanogel counterpart was included. Without the addition of CuCl_2_, none of the treatments showed apparent toxicity (Fig. 3e). Interestingly, both N-PDL1 and TN-PDL1 at the dose of 500 ng/ml effectively killed 4T1 cells when 10 μM CuCl_2_ was added (Fig. 3f), attributing to the function of copper ions in facilitating the nanogel escape from lysosomes,^28^ which is also evidenced in Fig. 2d. In contrast, both aPDL1 and polymer/copper at the same dose did not kill cancer cells. Since copper ions boost the efficacy of TN-PDL1, 10 μM CuCl_2_ was added in the following *in vitro* experiments. Furthermore, TN-IgG, loaded with a control antibody (isotype IgG), did not induce cell death (Fig. 3e–f), suggesting the safety of the carrier system.

In addition, TN-PDL1 also exhibited a cell-killing effect for NCI/ADR-Res, PANC-1, and MDA-MB-231 cancer cells (Supplementary Fig. 4), indicating the broad spectrum of its therapeutic effect. In contrast, TN-PDL1 is nontoxic for normal fibroblasts, NIH-3T3 cells (Supplementary Fig. 4). Since TN-PDL1 treatment downregulates the membrane expression of PD-L1 in cancer cells, we further evaluated the potential immunotherapeutic effect of TN-PDL1 *in vitro* by co-culturing 4T1 cells together with mouse peripheral blood mononuclear cells (PBMCs). It was revealed that the combination of TN-PDL1 and PBMCs exhibited a much stronger cell-killing effect than TN-PDL1 alone (Fig. 3g). This enhanced effect is even more significant when copper ions were included (Fig. 3h), suggesting a boosted immunotherapeutic effect.

### TN-PDL1 prevents the activation of STAT3 and inhibits the repair of DNA damage

To explore the mechanism of TN-PDL1 in killing cancer cells, we first investigated the effect of intracellular PD-L1 downregulation on STAT3 and pSTAT3. Fig. 3i–k revealed that the depletion of intracellular PD-L1 resulted in the reduction of both STAT3 and pSTAT3, which is critical for the proliferation and migration of cancer cells.^30^ It was also found that the TN-PDL1-induced protein reduction for STAT3, pSTAT3, PD-L1, and TRIM21 was dose-dependent, while the addition of CuCl_2_ further boosted its efficacy (Supplementary Fig. 3b–c). It has been reported that silencing PD-L1 prevents the repair of DNA damage.^31^ The accumulation of γ-H2AX, a marker for DNA damage, was observed in TN-PDL1 treated cancer cells (Fig. 3l–m), validating that TN-PDL1 inhibited DNA damage repair due to the depletion of intracellular PD-L1. The strong red signals accumulated in TN-PDL1 treated cells (Fig. 3n) further confirmed the inhibitory effect of TN-PDL1 in DNA damage repair.

### TN-PDL1 inhibits the migration and invasion of TNBC cells

**Fig. 4:**
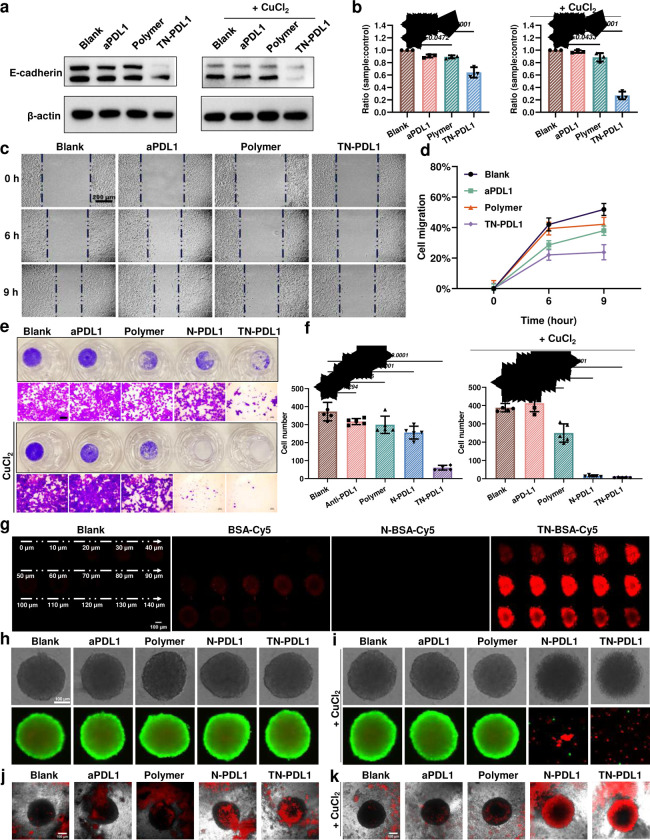
TN-PDL1 inhibits the migration and invasion of 4T1 cells, and promotes the disassembly of tumor spheroid and the infiltration of PBMCs. **a,** Protein expression level of E-cadherin in 4T1 cells after receiving aPDL1, polymer, and TN-PDL1 treatment with or without CuCl_2_ (10 μM) supplement for 16 h at an aPDL1 equivalent dose of 0.1 μg/mL. **b,** Quantitative analysis of E-cadherin expression in **a. c,** The representative would healing images of 4T1 cells after being treated with aPDL1, polymer, and TN-PDL1 with the supplement of 10 μM CuCl_2_. **d,** Cell migration distances in **c** over time. **e,** Representative images of the Transwell invasion after being treated with aPDL1, polymer, N-PDL1 and TN-PDL1 for 72 h. The cells were imaged after 1% crystal violet staining, magnification ×200. **f,** The number of invaded cells per visual field in **e**. Each well was imaged for 5 fields. Statistical analysis was performed via ANOVA one-way test, n=3, Mean ± SD, ****P < 0.0001. **g**, The penetration of different nanogels in 4T1 tumor spheroids. Tumor spheroids were treated with BSA-Cy5, N-BSA-Cy5, and TN-BSA-Cy5 for 4 h, and then imaged by a confocal microscope in z-stack from top to bottom. **h-i,** The disassembly capacity of TN-PDL1 on tumor spheroid. Tumor spheroids were treated aPDL1, polymer, N-PDL1, and TN-PDL1 treatments for 72 h with (**i**) or without (**h**) the supplement of 10 μM CuCl_2_ at an aPDL1 equivalent dose of 0.5 μg/mL. After the treatments, live/dead assay was used to visualize the disassembly and viability of the tumor spheroids. **j-k,** 4T1 Tumor spheroids were co-cultured with mouse PBMCs (red) together with the treatment described in **h** and **i**. The localizations of PBMCs in the tumor spheroid were recorded with a confocal microscope after 48 h of co-culture.

Recent research found that intracellular PD-L1 promotes epithelial-mesenchymal transition (EMT) in cancer cells.^25^ To probe the effect of TN-PDL1 on the EMT of 4T1 cells, western immunoblotting was employed to evaluate the expression of E-Cadherin, a marker for EMT, after TN-PDL1 treatment. Fig. 4a–b showed that TN-PDL1 coupled with CuCl_2_ downregulated the expression of E-Cadherin, suggesting the EMT repressive effect of TN-PDL1. Inspired by the STAT3 downregulation effect and EMT preventive effect of TN-PDL1, we further investigated how a low dose of TN-PDL1 affects the migration and invasion of 4T1 cells through wound healing assay and Transwell invasion assay, respectively. As expected, both N-PDL1 and TN-PDL1 effectively inhibited the migration (Fig. 4c–d) and invasion (Fig. 4e–f) of 4T1 cells. In contrast, free aPDL1 only showed a slightly inhibitory effect.

### TN-PDL1 promotes the disassemble of tumor spheroid and the penetration of PBMCs

To study if the functionalization of LBA could promote the penetration of nanogels into the tumor mass, tumor spheroid was employed. Bovine serum albumin (BSA) was adopted as a model protein to eliminate potential interference due to the activity of antibodies. Compared with free BSA and N-BSA, the ligand functionalized TN-BSA penetrated much deeper into the tumor spheroid (Fig. 4g). Encouraged by the outstanding tumor infiltrating capacity of the targeted nanogel, we further investigated the cell-killing effect of TN-PDL1 in a 3D tumor spheroid model. Without the supplementation of CuCl_2_, none of the treatments induced the death of 4T1 cells (Fig. 4h). Interestingly, the addition of CuCl_2_ resulted in the disassembly of the tumor spheroid (Supplementary Fig. 5a) and the death of the 4T1 cells (Fig. 4i), attributed to the better lysosomal escaping of TN-PDL1 when coupled CuCl_2_.

Since TN-PDL1 effectively attenuated the membrane expression of PD-L1 (Fig. 3c–d), we further probed if TN-PDL1 treatment could promote the penetration of PBMC into the tumor mass. Fig. 4j found that TN-PDL1 treatment alone induced the assembly of dye-prelabeled PBMCs surrounding the tumor spheroid. Interestingly, Fig. 4k revealed that the combination of TN-PDL1 and CuCl_2_ effectively promoted the penetration of PBMCs deep into the tumor spheroid, attributed to the reduced PD-L1 expression on the cell membrane (Supplementary Fig. 5b).

### TN-PDL1 biodistribution in a TNBC orthotopic tumor mouse model

**Fig. 5:**
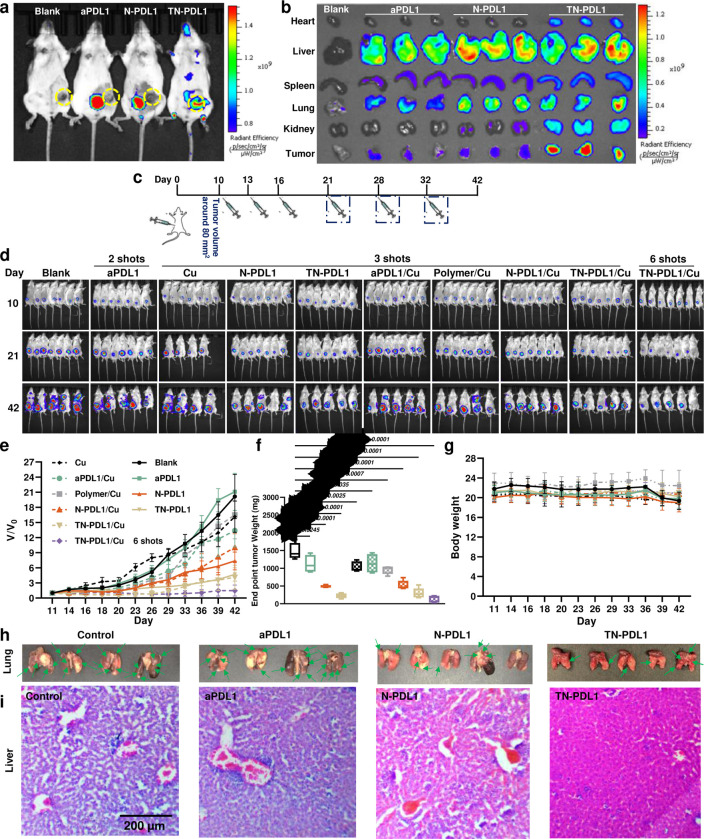
The tumor growth inhibitory effect of TN-PDL1 in 4T1 orthotopic breast cancer model. **a-b,** Biodistribution of TN-PDL1. *In vivo* (**a**) and *ex vivo* (**b**) images were obtained by IVIS Spectrum imaging system 4 h after i.v. administration of aPDL1-Cy5, N-PDL1-Cy5, and TN-PDL1-Cy5 nanogels. **c,** Timeline of tumor inoculation and treatment schedule. **d,** *In vivo* luminescence images of 4T1 orthotopic tumor during the course of treatment. Mice were treated at an aPDL1 equivalent dose of 2.5 mg/kg for aPDL1, N-PDL1, and TN-PDL1 via tail vein injection, and/or 2 mg/kg of copper gluconate (i.p.). **e,** The tumor volume change curve for different treatment groups. **f,** The tumor weight of different treatment groups at the endpoint. **g,** Mouse body weight change curve during after receiving different treatments. Statistical analysis was performed via ANOVA one-way test, n=3, Mean ± SD. **h,** Representative lung images from different treatment groups. Arrows indicate the metastatic tumors visible at the surface of the lungs. **i,** Representative histological images of liver from different treatment groups.

To investigate the biodistribution of the targeted nanogel, Cy5-labeled aPDL1 was employed. N-PDL1 and TN-PDL1 nanogels were i.v. injected into BALB/c mice bearing orthotopic 4T1 TNBC tumor established following our published protocol and detected with an IVIS imaging system.^32^ It was revealed that free aPDL1 and N-PDL1 were quickly cleared from the circulation system, as evidenced by the strong fluorescence signal in the bladder 4 h post injection (Fig. 5a). In contrast, a weak fluorescence signal was observed in the bladder of TN-PDL1 treated mice, suggesting extended circulation time for TN-PDL1. The *ex vivo* image shown in Fig. 5b revealed that TN-PDL1s were selectively retained in the tumor tissue, as evidenced by the brightest fluorescence signal, which was much stronger than that from the N-PDL1 treated group, indicating that the functionalization of LBA did boost nanogel targeting TNBC tumor. The overall stronger fluorescence signals found in other organs further confirmed that LBA modification prolonged the circulation of TN-PDL1, which subsequently resulted in its more efficient tumor-targeting effect.

### TN-PDL1 inhibits the growth of TNBC

The tumor growth inhibitory effect of the TN-PDL1 was evaluated in BALB/c mice inoculated with 4T1-Luc cells orthotopically. Treatments were given via tail vein injection on day 10, 13, and 16 post-cell inoculation at an aPDL1 equivalent dose of 2.5 mg/kg with/without the i.p. administration of copper gluconate at the dose of 2 mg/kg (Fig. 5c). For aPDL1 treatment, only the first two doses were given to four mice, because the other three mice immediately died upon a third dose, possibly due to intrinsic toxicity and/or a xenogeneic hypersensitive response to rat-derived aPDL1.^33^ To study the benefit and potential side effects of long-term treatment, one group of mice received an additional three doses of TN-PDL1 therapy on day 21, 28, and 32. Fig. 5d–f revealed that free aPDL1 treatment only slightly inhibited tumor progression at the dose of 2.5 mg/kg. In contrast, both N-PDL1 and TN-PDL1 significantly inhibited tumor growth. At the same time, TN-PDL1 exhibited a much greater inhibitory effect than N-PDL1, likely attributable to the cancer cell-targeting effect of LBA (Fig. 5a–b and Supplementary Fig. 6–7). It is worth noting that the addition of copper did not result in a notable difference in the treatments involved N-PDL1 and TN-PDL1, which is different from the *in vitro* results shown in Fig. 3e–h. We postulate this may be due to the elevated copper concentration in the tumor tissue,^34^ which negates the necessity of copper supplement *in vivo*. Furthermore, six doses of TN-PDL1 treatment exhibited a more significant inhibitory effect on tumor progression than three doses (Fig. 5d–f).

### TN-PDL1 inhibits the metastasis of TNBC without inducing side effects

The decrease in tumor nodules in the lungs of TN-PDL1 treated mice revealed that TN-PDL1 also attenuated the metastasis of the TNBC to the lung (Fig. 5h and Supplementary Fig. 7), which may be attributed to the anti-migration and anti-invasion effect of TN-PDL1 (Fig. 4c–f). In contrast to the TNBC tumor metastasis to the liver in other treatment groups, an attenuated liver metastatic effect was also observed in TN-PDL1 treated mice (Fig. 5i). More importantly, animals in all treatment groups did not see apparent weight loss except the control group, in which mice lost about 13% weight at the end of the study (Fig. 5g). In addition, no abnormal structure was observed in the major organs among all groups (Supplementary Fig. 8).

### TN-PDL1 effect on the expression of PD-L1 and pSTAT3 in the tumor tissue

**Fig. 6:**
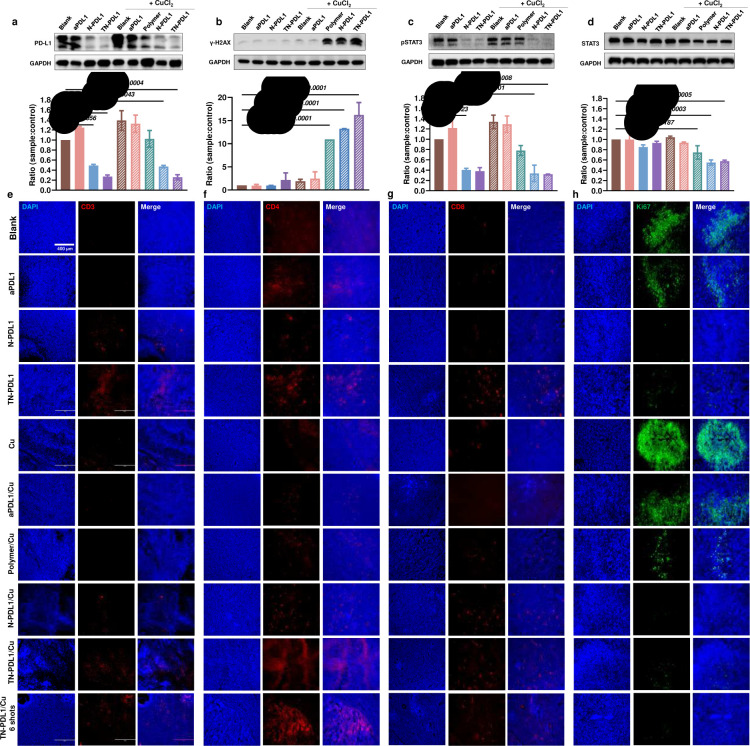
TN-PDL1 inhibits PD-L1 expression and promotes T cell infiltration in the tumors. Protein expression of **a,** PD-L1, **b,** γ-H2AX, **c,** pSTAT3, and **d,** STAT3 in the tumor tissues after two doses of treatment, (n=2). Statistical analysis was performed via ANOVA one-way test, n=3, Mean ± SD. The endpoint harvested tumor sections were detected for T cell subpopulations of **e,** CD3^+^ (red), **f,** CD4^+^ (red), **g,** CD8^+^ (red), and cell proliferation maker **h,** Ki-67 (green) by immunohistochemistry. The nuclei were labeled with Hoechst 33342 (blue). Scale bars = 400 μm.

To investigate how TN-PDL1 treatment affects the expression of PD-L1, γ-H2AX, pSTAT3, and STAT3 *in vivo*, two mice were sacrificed 3 days after receiving the second treatment dose. Western immunoblotting found that TN-PDL1 treatment reduced the PD-L1, pSTAT3, and STAT3 expression in the tumor tissue to 32%, 30%, 56% (Fig. 6a, 6c–d), respectively, proving the success of the application of Trim-Away *in vivo*. As expected, the expression of γ-H2AX was upregulated by 15.5 folds in the tumor after TN-PDL1 treatment (Fig. 6b).

### TN-PDL1 regulates the infiltration of T cells inside the tumor tissue

TN-PDL1 downregulated PD-L1 expression on the membrane of cancer cells and induced better PBMCs penetration in the tumor spheroid *in vitro* (Fig. 4k). To probe whether TN-PDL1 could result in a greater T cell infiltration *in vivo*, T cells resident within the tumor tissues were detected by immunohistochemistry. In addition to more CD3^+^, CD4^+^, and CD8^+^ T cells in the edge of the TN-PDL1 treated tumor (Supplementary Fig. 9), Fig. 6e–g revealed that more CD3^+^, CD4^+^, and CD8^+^ T cells were attracted to the center of the tumor mass after TN-PDL1 treatment. This suggests that the TN-PDL1 induced decrease in the expression of PD-L1 on cancer cell membrane promoted the infiltration of overall T cells, T helper cells, and cytotoxic T cells. In contrast, little or no T cells were detected in the tumor tissues of control and aPDL1-treated animals. Consequently, much fewer Ki67-stained proliferating cells were detected in the center region of the tumors from the TN-PDL1 treated group (Fig. 6h).

## Discussion

Trim-Away is a novel and versatile approach for the degradation of intracellular proteins. However, due to the lack of an effective *in vivo* antibody delivery approach, till now, Trim-Away can currently only be realized in oocytes/zygotes and used for the study of gene/protein function in embryo development. With the help of our developed Nano-ERASER technology, we successfully delivered a model antibody, aPDL1, into cancer cells via TN-PDL1, and induced the reduction of total and membrane PD-L1 levels through Trim-Away. As a result, when TN-PDL1 was coupled with copper ions, the depletion of PD-L1 induced the downregulation of E-Cadherin, STAT3, and pSTAT3 and inhibited DNA damage repair capacity, which altogether impeded the proliferation, migration, and invasion of 4T1 cells. In addition, the reduced membrane PD-L1 expression promoted T cell infiltration for cancer immunotherapy. Benefiting from incorporating a tumor-targeting ligand, TN-PDL1 was accumulated in the tumor tissue in an orthotopic TNBC mouse model. Consequently, TN-PDL1 inhibited primary tumor growth and prevented lung and liver metastasis. Unlike the clinically used free aPDL1, TN-PDL1 was safe for multi-dose administration.

This study showed a successful *in vivo* Trim-Away treatment in adult animals for the first time and proved that Nano-ERASER is an effective tool for cancer treatment. Furthermore, due to the interchangeable nature of the antibody and targeting ligand in the Nano-ERASER, we expect many diseases caused by the malfunction of proteins, including undruggable targets, could be effectively controlled through Trim-Away. Furthermore, the success of *in vivo* Trim-Away through Nano-ERASER technology would broaden the application for antibodies and provide a highly effective and specific tool for studying gene/protein function in adult animals.

## Supplementary Material

Supplement 1
