# Supplementary material for: Trim-Away in adult animals through Nano-ERASER and its application in cancer therapy
Source: Res Sq. 2023 Jan 17:rs.3.rs-2298306. Preprint. [Version 1] doi: 10.21203/rs.3.rs-2298306/v1 (PMC9882598; doi:10.21203/rs.3.rs-2298306/v1)
Supplement: Supplement 1 [file NIHPPrs2298306v1-supplement-1.pdf]

Supplementary Materials:

Trim-Away in adult animals through Nano-ERASER and its application in cancer therapy

Mingming Wang[1], Junfeng Wang[2], Yuzhen Wang[2], Manikanda Keerthi Raja[3], Gourab Gupta[3], Xiangxiang Hu[1], Shanshan Shi[1], Hexin Chen[3], Daping Fan[2], and Peisheng Xu[1,]*

Materials and Methods

Materials

Pierce™ Rapid Gold BCA Protein Assay Kit, Fetal bovine serum (FBS), Gibco Dulbecco's modified Eagle medium (DMEM), trypsin-EDTA free (0.25%), penicillin-streptomycin (PS), Corning® 96-well Black/Clear Round Bottom Ultra-Low Attachment Spheroid Microplate, Molecular Probes™ Cell Imaging Kit, Ethidium homodimer-1 (EthD-1), SuperSignal™ West Pico PLUS Chemiluminescent Substrate, and Corning Transwell® Permeable supports 6.5 mm Insert, 24 well plate with 8.0 μm Polycarbonate membrane were purchased from Thermo Fisher Scientifc, Inc. (Waltham, MA, USA). Hoechst 33342 was purchased from Invitrogen. BioAcryl-P (30%, 37.5:1) liquid was acquired from Alfa Aesar. (3-(4,5-dimethylthiazol-2-yl)-2,5-diphenyltetrazolium bromide (MTT) was purchased from Tokyo Chemical Industry Co., Ltd (Portland, OR, USA). EDTA-free mini protease inhibitor tablet was supplied by Roche Applied Science (Indianapolis, IN, USA). D-luciferin potassium salt was purchased from PerkinElmer, Inc. (Waltham, MA, USA). Anti-PD-L1 antibody and control IgG were purchased from Bio X Cell. Cyanine 3 NHS ester and sulfo-Cy5 NHS ester were purchased fromLumiprobe. LysoTracker Green DND-26 and Cell tracker™ Deep Red were purchased from Life Technologies. CellBrite® Steady Membrane Labeling Kit was purchased from Biotium. All other chemical reagents and solvents used in this research were purchased from Sigma-Aldrich Chemical Co. (St. Louis, MO, USA) and used directly without further process unless

specially noted. PDA-PEG, LBA-PDA-PEG, PDA-PEG-BME, and PDA-PEG-NPC polymers were prepared according to our published methods.[12]

Cell culture

4T1, MDA-MB-231, PANC-1, NIH-3T3, and NCI-ADR/Res cells were purchased from ATCC. The cells were cultured in Dulbecco's Modified Eagle's Medium (DMEM) containing 10 % of fetal bovine serum (FBS, Gibco), 100 U/mL of penicillin, and 100 µg/mL of streptomycin under a humidified atmosphere of 5 % CO_2 at 37 ℃. The culture medium was replaced with a fresh one every two days.

Synthesis of PDA-PEG-aPDL1 and PDA-PEG-IgG

PDA-PEG-aPDL1 and PDA-PEG-IgG were synthesized according to our published method for polymer-antibody conjugate except for replacing anti-GFP antibody with aDPL1 and IgG, respectively.[12] The resulted polymer-antibody conjugate was purified through dialysis in a Spectra/Por® dialysis tube (regenerated cellulose, MWCO: 100 kDa) for 48 h against PBS buffer at 4 °C.

Fabrication of TN-PDL1 and TN-IgG nanogels

LBA-PDA-PEG (50 mg, 30% LBA conjugation density) was dissolved in 4 mL of DMSO. After that, PDA-PEG-aPDL1 (50 mg, contains aPDL1 10 mg) PBS solution was added dropwise at room temperature under stirring. Five minutes later, 4.605 mg (tris(2-carboxyethyl)phosphine (TCEP) dissolved in 1 mL of DMSO was added dropwise under stirring. The mixture was kept at room temperature (RT) for 15 min to initiate the crosslinking process. After that, the mixture solution was added dropwise into 50 mL dd H_2O at 4 °C under stirring. Then the produced nanogels were purified through dialysis. The final nanogels were stored in PBS (pH 7.4) at 4 °C before use. For the preparation of non-targeted nanogel, LBA-PDA-PEG was replaced with PDA-PEG (50 mg) and fabricated following the protocol described above. For the preparation of TN-IgG, aPDL1 was replaced with IgG and fabricated following the TN-PDL1 protocol described above.

Characterization of the nanogels

The morphology of the TN-PDL1 was characterized by transmission electron microscope (Hitachi HT7800 TEM, Hitachi High Technologies, Tokyo, Japan), while the hydrodynamic size and zeta potential of TN-PDL1 were measured by Nano ZS Zetasizer (Malvern Instruments, UK). The successful conjugation of antibody to the polymer and the redox response release were confirmed by 8% sodium dodecyl sulfate-polyacrylamide gel electrophoresis (SDS-PAGE) and stained with Coomassie brilliant blue (Invitrogen, Oregon, USA) method. After that, the PAGE-gel was washed with dd H_2O until the background was clear. ChemiDoc™ imaging system (BIO-RAD) was used to record the gels.

Cellular uptake of the nanogel in cancer cells

To investigate the cellular internalization of the nanoparticles, 4T1 cells were cultured in 35 mm glass-bottom dishes at a density of 5,000 per well. After 24 h of culture, the old medium was replaced with a fresh one containing Cy5 (final concentration 5 µg/mL) labeled aPDL1, N-PDL1, and TN-PDL1. After 3 hours, a diluted CellBrite® Steady dye (1:1000) was added to all treatments to stain the cells for 1 h. Cells were washed with PBS first, then replaced with fresh medium. The fluorescence images were observed with a Carl Zeiss LSM700 confocal microscope, where the red signal indicated the uptake of aPDL1 and the green signal showed the cell membrane. Flow cytometry was further used to quantitatively measure the internalization of aPDL1 into 4T1 cells.

Subcellular localization of targeted nanogel and intracellular release of antibody

4T1 cells were cultured in 35 mm glass-bottom dishes at a density of 5,000 per well. After 24 h of culture, the old medium was replaced with a fresh one containing TN-IgG-Cy5 for 3 h with or without the addition of 10 µM $CuCl_2$. Lysotracker green DND-26 (1:1000) was added and incubated for 30 min before the observation with a confocal microscope to track the subcellular localization of the targeted nanogels. To investigate the intracellular release of the antibody, the control antibody (IgG) and the polymer were pre-labeled with Cy3 and Cy5, respectively,

before the fabrication of a Cy3/Cy5 dual-labeled nanogel (TN-IgG-Cy3-Cy5). 4T1 cells were incubated with TN-IgG-Cy3-Cy5 and then observed under a confocal microscope upon the excitation of a 555 nm laser (emission: 558-610 nm and 640-700 nm) at 4 h, 8 h, and 24 h.

In vitro cytotoxicity assay

Cells were seeded in 96-well plates (8,000 cells/well) for 24 h prior to the study. Different concentrations of aPDL1, N-PDL1, TN-PDL1, IgG, and TN-IgG in a culture medium were added to the above cells, supplementing with or without $CuCl_2$ (10 μM). The cells were then incubated for 24 h or 72 h in 95/5% air/CO_2 at 37 °C and followed by MTT assay. To evaluate the impact of PBMCs on the cytotoxicity of TN-PDL1, PBMCs isolated from BALB/c mice were added with the different treatments to the 4T1 cells under the ratio of 10:1 (PBMCs:4T1). After that, the cells were incubated for another 24 h before the addition of MTT reagent.

Immunoblotting

4T1 cells were harvested after receiving different treatments and lysed on ice in a RIPA buffer containing 1 % cocktail protease inhibitor. Proteins were separated by 8 % of SDS-PAGE electrophoresis and transferred onto a PVDF membrane. After being blocked with 5 % of fat-free dry milk solution in 1× TBST (1× TBS and 0.1% Tween 20) for 1 h, the membrane was incubated with primary antibodies against PD-L1 (BioXCell, cat. no. BE0101, 1:1000), STAT3 (Cell Signaling, cat. no. 12640S, 1:1000), pSTAT3 (Cell Signaling, cat. no. 9145T, 1:1000), TRIM21 (Proteintech, cat. no. 12108-1-AP, 1:1000), GADPH (Sigma life science, cat. no. G9545, 1:2000), γ-H2AX (Proteintech, cat. no. I0856-i-AP, 1:1000), E-cadherin (Proteintech, 20874-I-AP, 1:1000), and β-actin (1:1000) overnight at 4 °C, respectively. After that, the membranes were washed with TBST and incubated with the secondary antibodies for 1 h at RT. Enhanced chemiluminescence was used to detect the expression of the targeted protein under the ChemiDocTM imaging system (BIO-RAD). β-actin and GADPH were used as internal reference proteins.

Immunocytochemistry

The 4T1 cells were cultured in a quarter dish with a glass bottom (Greiner Bio-One, cat. no. 627870) overnight. Different treatments at an aPDL1 equivalent concentration of 100 ng/mL were added to each well and incubated for 24 h. After that, cells were washed with PBS every 5 min for 3 times, and 4% paraformaldehyde was used to fix the cell at RT for 30 min. The cells were then treated with 0.1% Triton-100 (TX-100) for 2 min, and the non-specific binding was blocked by 5% BSA for 1 h in RT. The primary antibodies of anti-PDL1 (1:100) and anti-γ-H2AX (1:200) were incubated at 4 C° overnight. The cells were washed by 1× PBST (1× PBS and 0.1% Tween 20) for 5 times and followed by incubating with the secondary antibodies goat anti-rat IgM-488 (abcam, cat. no. ab98368, 1:500) and Alexa Fluor 633 goat anti-rabbit IgG (Life technologies, cat. no. A21070, 1:2000) for 1 h at RT. Finally, the cell nuclei were stained with Hoechst 33342 (Invitrogen, cat. no. 911741, 1:2000). The images of the cells were recorded by a fluorescence microscope (EVOS^{TM} FL, ThermoFisher Scientific, MA, USA).

Wound healing assay

4T1 cells were seeded in 6-well plates at the density of 2,000,000 cells/well. When the cell confluency reached almost 100%, the supernatant was aspirated, and then the cells were scratched with a 200 µL pipette tip to generate the wound. After being washed with PBS, the cells were incubated with a medium containing 50 ng/mL aPDL1, N-PDL1, and TN-PDL1 with or without the supplement of 10 µM $CuCl_2$ at 3, 6, and 9 h. The scratched areas were monitored and photographed with light microscopy.

Cell Invasion assay

Cell invasion assay was performed using a 24-well plate with 8 µm pore-size Transwell inserts (Costar Corp., Cambridge, MA, cat. No. 3342). Briefly, 50 µL Matrigel was added into each insert and solidified at 37 °C for 30 minutes to generate a thin gel layer. 4T1 cells treated with 50 ng/mL of aPDL1, N-PDL1, TN-PDL1 in 200 µL serum-free medium with or without the supplement of 10 µM $CuCl_2$ were transferred into upper chambers at the concentration of 100,000 cells/well. The bottom chambers were filled with 600 µL complete medium with the same concentration of respective drugs. After being incubated at 37 °C for 72 h, cells in the

upper chamber were removed, and the invaded cells were fixed with 4% paraformaldehyde and stained with 1% crystal violet before being counted under a light microscope.

Tumor spheroid penetration assay

To investigate the effect of TN-PDL1 on the infiltration of T cell in tumor tissue, tumor spheroid model was adopted. Briefly, 4T1 cells were seeded in 96-well ultra-low-attachment (ULA) plates (Corning, MA, USA) and incubated at 37 °C for 3 days or till the formation of tumor spheroids. Various treatments were added, including aPDL1, N-PDL1, TN-PDL1, and TN-IgG in a culture medium supplemented with or without $CuCl_2$ (10 μM). PBMCs stained with CellTracker™ Deep Red Dye (Invitrogen, C34565, 1:1000) were added to the medium to probe its tumor infiltration effect. In parallel, PBMCs without dye staining were added to investigate the cytotoxicity of the combination of TN-PDL1 and PBMCs. The morphology of the tumor spheroids was observed and imaged using a fluorescence microscope every 24 h using an EVOS^{TM} FL microscope, while the penetration of the PBMCs was imaged using a confocal microscope (Carl Zeiss LSM700) under z-stack mode.

Orthotopic breast cancer model

All animal experiments were carried out following the protocol approved by the Institutional Animal Care and Use Committee (IACUC) of the University of South Carolina. Female BALB/c mice were purchased from Jackson laboratory. For tracking the tumor growth, 4T1 cells stably expressing luciferase (4T1/Luc2) were adopted. Orthotopic breast cancer model was established by injecting 4T1/Luc2 cells (2 X 10^5 in 20 μL PBS) into the right 4th mammary fat pad of 6~8 weeks old mice, and the primary tumor growth was monitored through bioluminescence signal using IVIS imaging system following the method described in the literature.[32]

In vivo distribution of nanoparticles

To track nanoparticles *in vivo* distribution in the above prepared 4T1 orthotopic tumor-bearing BALB/c mice, Cy5 was loaded in the nanogels as a fluorescence probe by conjugating with

aPDL1. Cy5 labeled aPDL1, N-PDL1, and TN-PDL1 were injected intravenously at a Cy5 dose of 0.5 mg/kg. Three hours post-injection, the mice were anesthetized and imaged using an IVIS Lumina III imaging system (excitation: 620 nm; emission: 690 nm). After that, the animals were sacrificed, and the major organs, including heart, liver, spleen, lung, kidney, and tumor, were collected for *ex vivo* imaging to investigate the tissue distribution of aPDL1 and aPDL1 loaded nanogels.

Antitumor therapy in the orthotopic breast cancer model

The above-established tumor-bearing mice were randomly divided into 9 groups when the primary tumors reached 50 mm^3, including saline, aPDL1, empty polymer nanogel, N-PDL1, and TN-PDL1 with or without the supplement of 2 mg/kg of copper gluconate (i.p.). Animals received three doses of corresponding treatments at an aPDL1 equivalent dosage of 2.5 mg/kg on days 10th, 13th, and 16th post the inoculation of the cancer cells via tail vein administration except for the aPDL1 treatment, in which only the first two doses were given to 4 mice because the other 3 mice immediately died upon the administration of the third dose, possibly due to intrinsic toxicity and/or xenogeneic hypersensitive response to rat-derived aPDL1. In addition, the progression of the primary tumor and lung metastases was monitored by measuring the size and bioluminescence imaging.

Histology and immunohistochemistry analysis

The harvested tumor tissues and major organs were fixed with 4% paraformaldehyde for 24 h, followed by 15% sucrose (24h)/30% sucrose (24h), and processed by OCT embedding and cryosectioning. Tissue sections (heart, liver, spleen, and kidney) and lung sections were hematoxylin and eosin (H&E) stained for acute toxicity evaluation and lung metastasis evaluation. The tissue sections were attached to poly-L-lysine-coated slides and warmed to RT for 30 min. After that, the OCT was washed with PBS, and the slices were dried at RT before blocked with 5% BSA for 1 h. The primary antibodies of CD3 (Proteintech, cat. no. 17617-1-AP, 1:100), CD4 (Proteintech, cat. no. 19068-1-AP, 1:100), CD8 Alexa Flour® 647 (Santa Cruz Biotechnology, cat. no. sc-18860, 1:100), and Ki67 (Cell Signaling, cat. no.

11882S, 1:500) were incubated with the slides at 4 C° overnight followed by detecting with Alexa Fluor 633 goat anti-rabbit IgG (Life Technologies, cat. no. A21070, 1:2000) except CD8 for 1 h at room temperature. Then, the nuclei of the cells were detected by Hoechst 33342 (Invitrogen, cat. no. 911741, 1:2000). The sections were dried under RT and sealed by ProLongTM Gold antifade reagent (Invitrogen, cat. no. P36734) with the cover glass. All the immunofluorescence images were recorded with a fluorescence microscope (EVOS^{TM} FL, ThermoFisher Scientific, MA, USA).

Statistical analysis

All data were displayed as mean ± standard deviation (SD) (n≥3), and the statistical significance was analyzed by GraphPad Prism 9.0 (GraphPad Prism Software Inc., San Diego, California) using Student's t-test or ANOVA with Tukey's significant. Differences were considered significant when the p-value was less than 0.05.

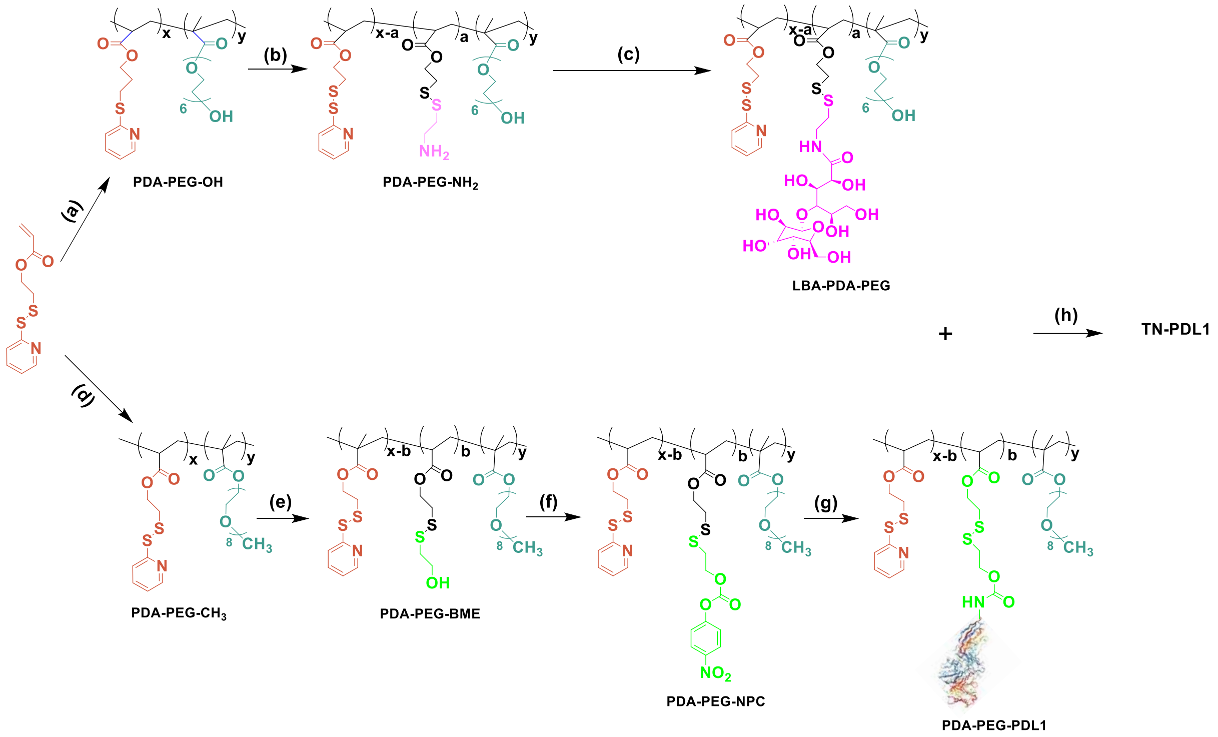

Supplementary Scheme. 1. The scheme for the synthesis of TN-PDL1 nanogel. (a) Poly (ethylene glycol) methacrylate (PEG_{360}), 2’2-azobisisobutyronitrile (AIBN), anisole, 65 ℃. (b) Cysteamine, dichloromethane, r.t. (c) Lactobionic acid, 1-Ethyl-3-(3-dimethylaminopropyl)carbodiimide hydrochloride, N-hydroxy succinimide, methanal, dichloromethane, r.t. (d) Poly(ethyleneglyco)methyl ether methacrylate (PEG_{500}), 2’2-azobisisobutyronitrile (AIBN), anisole, 65 ℃. (e) 2-Mercaptoethanol (BME), dichloromethane, r.t. (f) 4-Nitrophenyl Chloroformate, pyridine, dichloromethane, 4℃ shift to r.t. (g) Anti-PD-L1, PBS buffer (pH=8.4), dimethyl sulfoxide (DMSO), 4℃. (h) Tris(2-carboxyethyl)phosphine Hydrochloride (TCEP), dimethyl sulfoxide (DMSO), r.t.

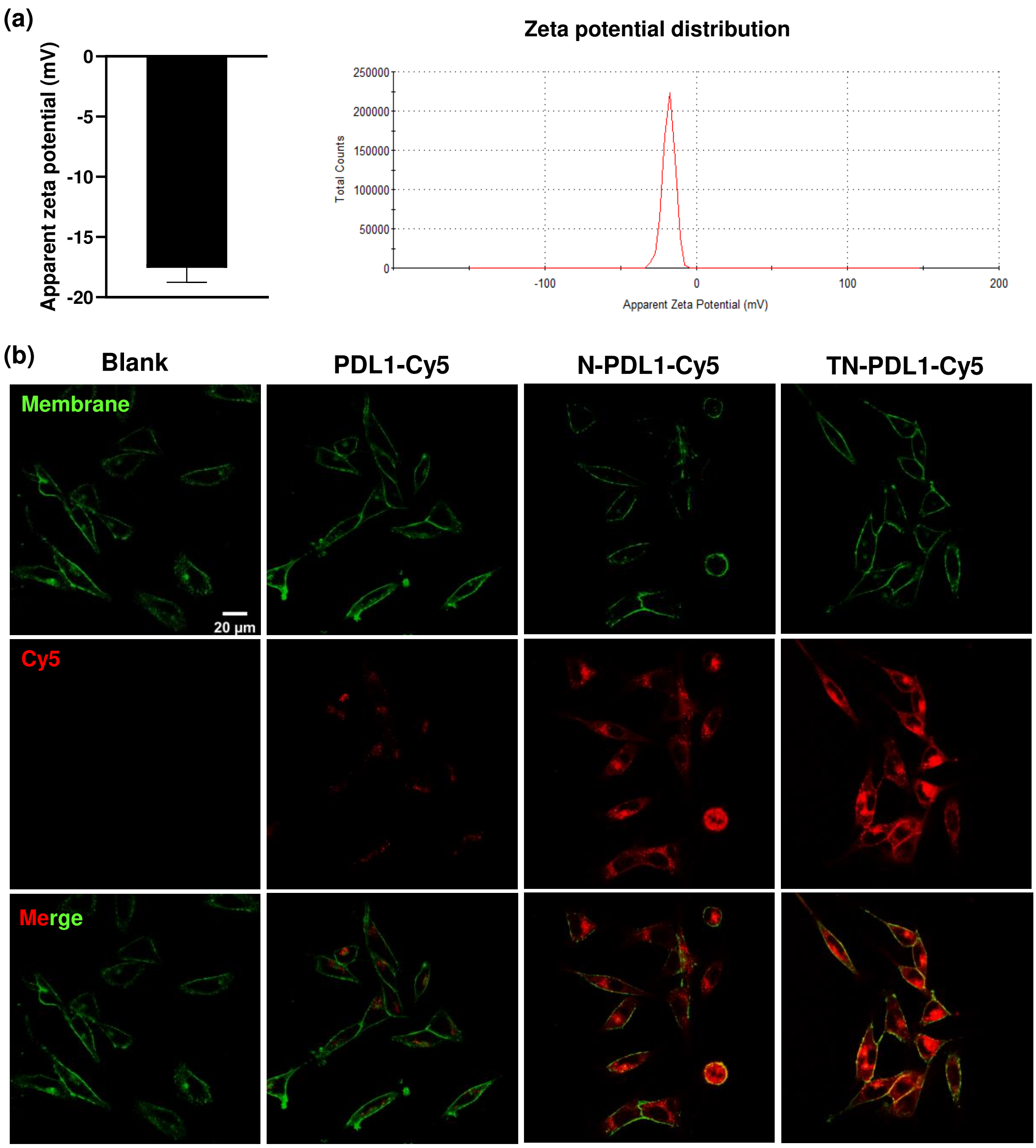

Supplementary Fig. 1. Zeta potential distribution of TN-PDL1 (a) and the cellular uptake of TN-PDL1 (b) by 4T1 cells. The TN-PDL1 nanogels were dispersed in dd H_2O (**a**). (**b**) Fluorescence images of 4T1 cells treated with different Cy5-labeled (red) nanoparticles for 4 h. The nuclei were stained by Hoechst 33342 (blue). CellBrite® Steady Membrane Labeling Kit was used to label the cell membrane. The scale bar was 20 μm.

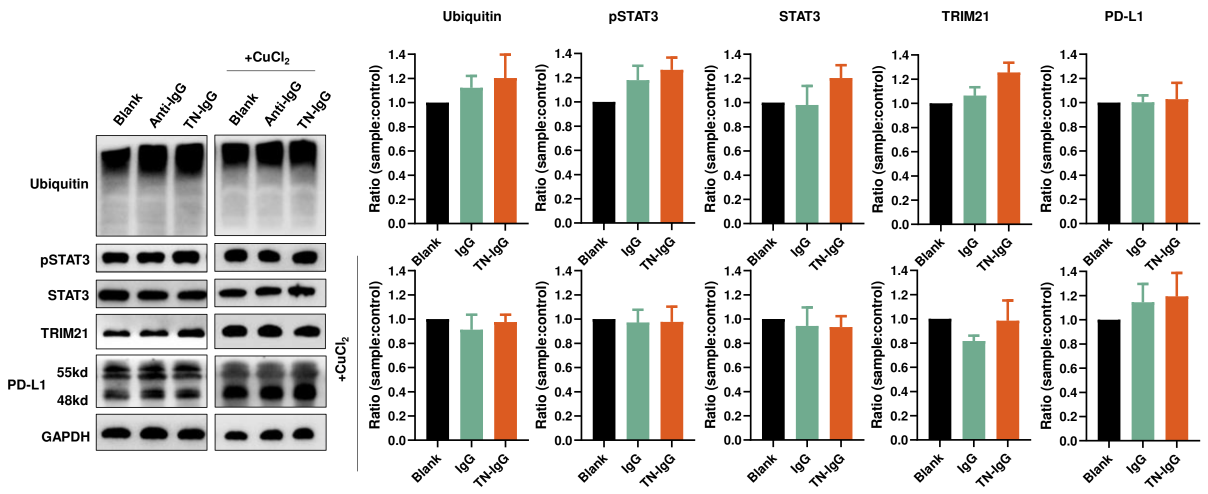

Supplementary Fig. 2. The protein expression level of 4T1 cells treated with TN-IgG. 4T1 cells were treated with free IgG and TN-IgG at the IgG equivalent dose of 0.25 μg/mL with or without 10 μM $CuCl_2$ for 16 h. Quantitative graphs of the intensity for ubiquitin, pSTAT3, STAT3, TRIM21, and PD-L1 were expressed as Mean ± SD, n=3.

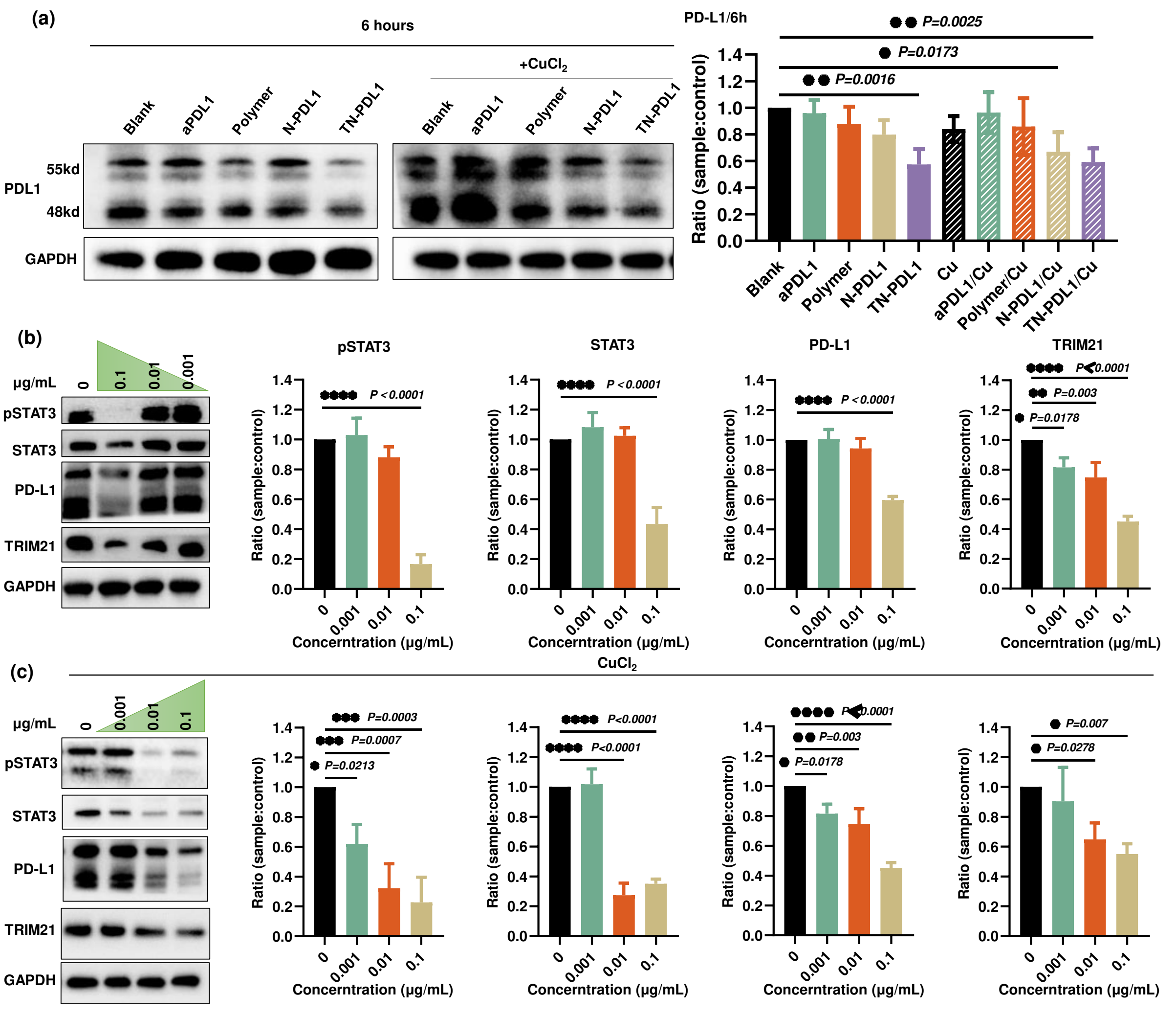

Supplementary Fig. 3. 4T1 cell protein suppression effect of different treatments by western blot assays. (a) Protein expression and quantitative analysis of PD-L1 in 4T1 cells after receiving aPDL1 (0.1 μg/mL), N-PDL1, and TN-PDL1 treatment with or without CuCl2 (10 896 μM) supplement for 6 h. (b) Protein expression and quantitative analysis of Ð-L1, STAT3, pSTAT3, and TRIM21 after receiving different concentrations of TN-PDL1 treatment with (c) and without (b) 10 μM $CuCl_2$ supplement for 16 h. The quantitative analysis was performed through ANOVA one-way test, n=3, Mean ± SD, ****$P < 0.0001$.

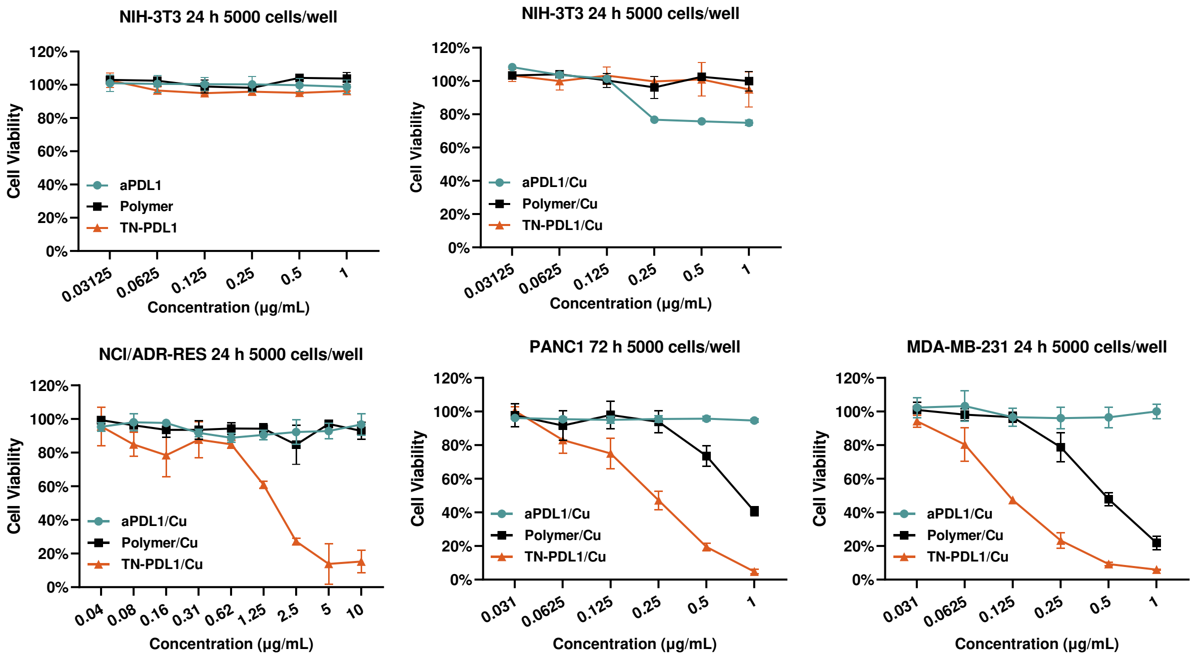

Supplementary Fig. 4. The viability of NIH-3T3, NCI/ADR-RES, PANC1, and MDA-MB-231 cells after being treated with TN-PDL1. The viability of the cells were evaluated with MTT assay. Data were expressed as Mean ± SD, n=3.

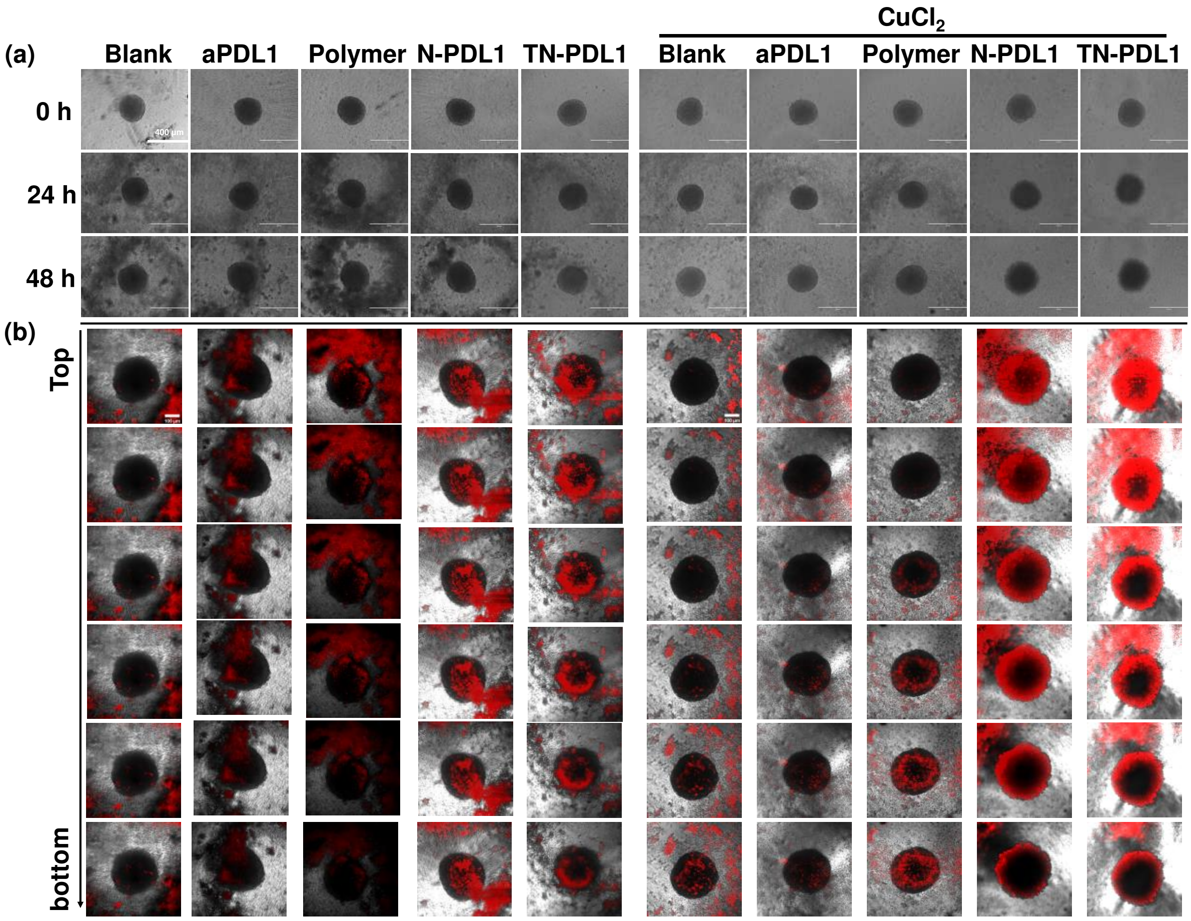

Supplementary Fig. 5. The images tumor spheroid (a) and the infiltration of PBMCs in the tumor spheroid (b) after being treated with TN-PDL1. Representative images of 4T1 tumor spheroids after being treated with aPDL1, control polymer, N-PDL1, and TN-PDL1 with or without 10 μM $CuCl_2$ supplement for 0, 24, and 48 h (**a**). The scar bar was 400 μm. PBMCs were pre-labeled with cell-tracker red (633) and simultaneously added with the above treatments (b). The confocal z-stack projections show the penetration of the PBMCs from the top to the bottom of the 4T1 tumor spheroids. The scale bar was 100 μm.

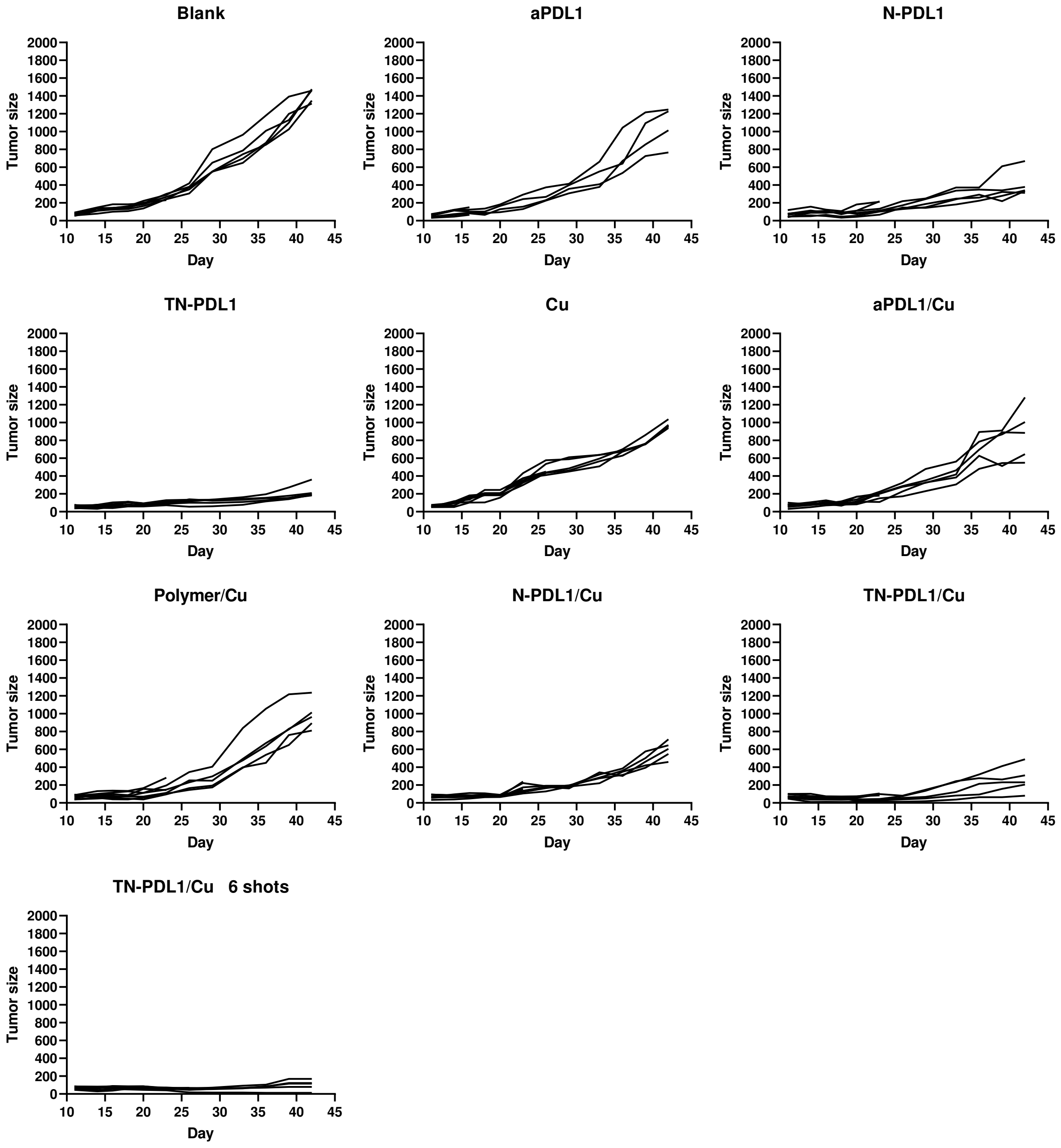

Supplementary Fig. 6. Tumor growth curve. The size of the 4T1 primary orthotopic tumor during the course of treatment. Mice were treated at an aPDL1 equivalent dose of 2.5 mg/kg for aPDL1, N-PDL1, and TN-PDL1 via tail vein injection, and/or 2 mg/kg of copper gluconate (i.p.).

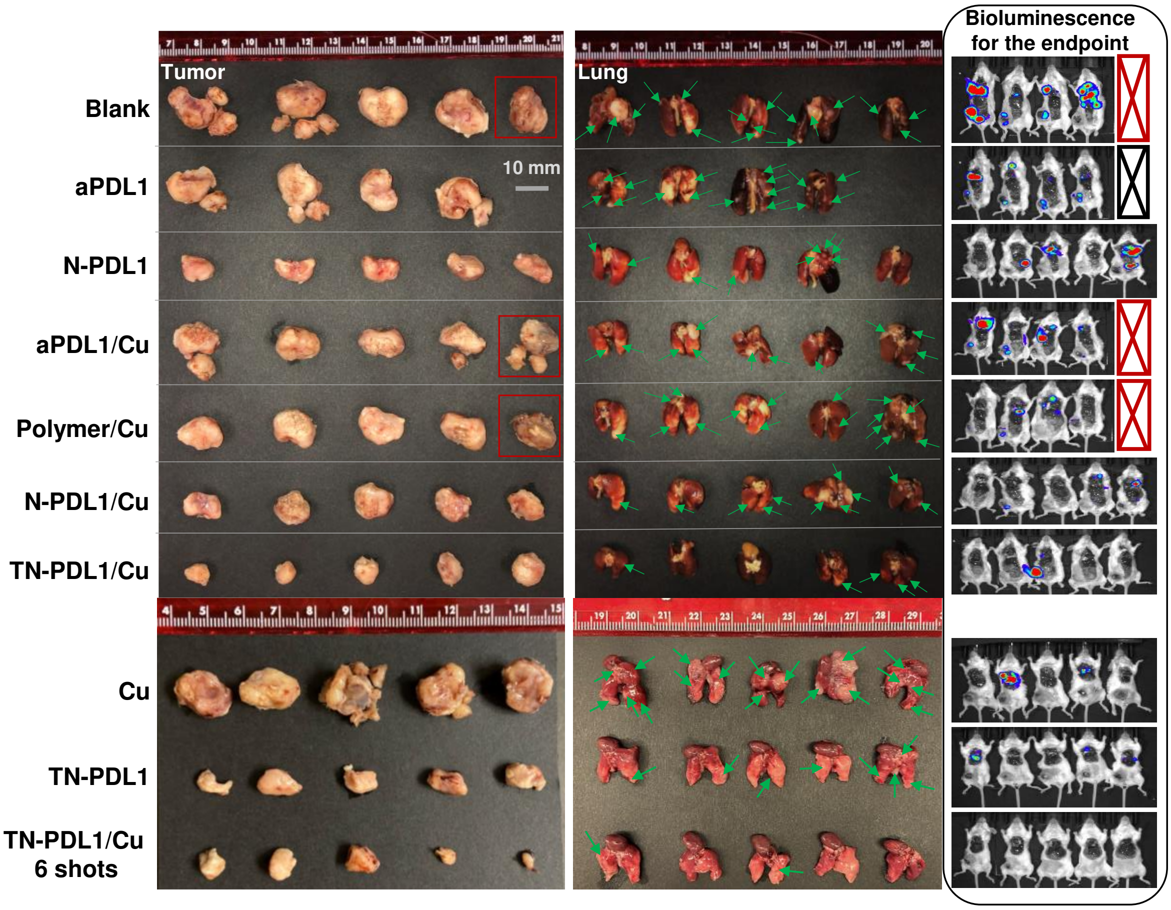

Supplementary Fig. 7. The images of the primary tumors and lungs. The primary tumors (left panel) and lungs (middle panel) were collected from different treatment groups at the endpoint of the experiment. Animals were sacrificed at day 42 after being injected (i.p) with luciferin (100 µg/mice), and the body cavities were opened and subjected to IVIS observation (right panel). Green arrows indict the tumor nodules in the lung. The red boxes indicate that those animals reached endpoint earlier than day 42.

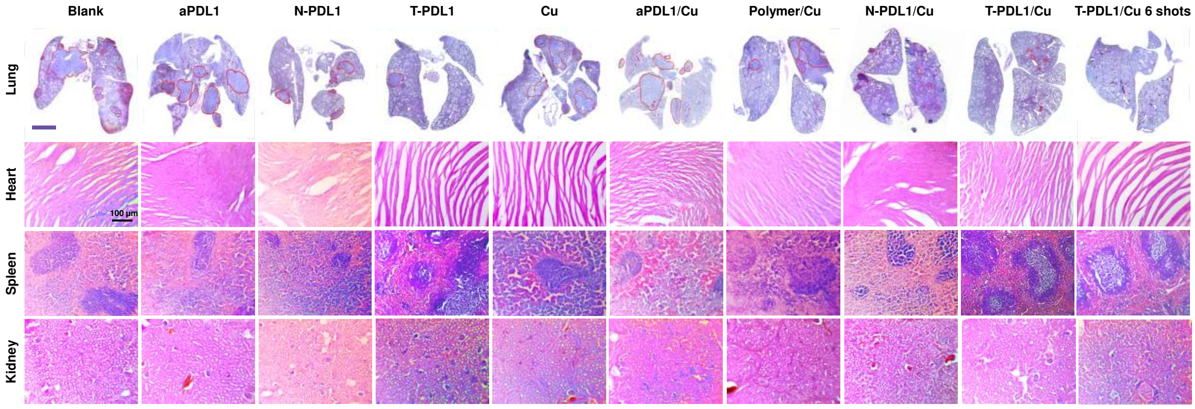

Supplementary Fig. 8. Representative images of HE stained tissue sections. Red lines circled the metastasis nodules in the lung tissues. Scale bars were 2000 μm and 100 μm for top panel and bottom panel, respectively.

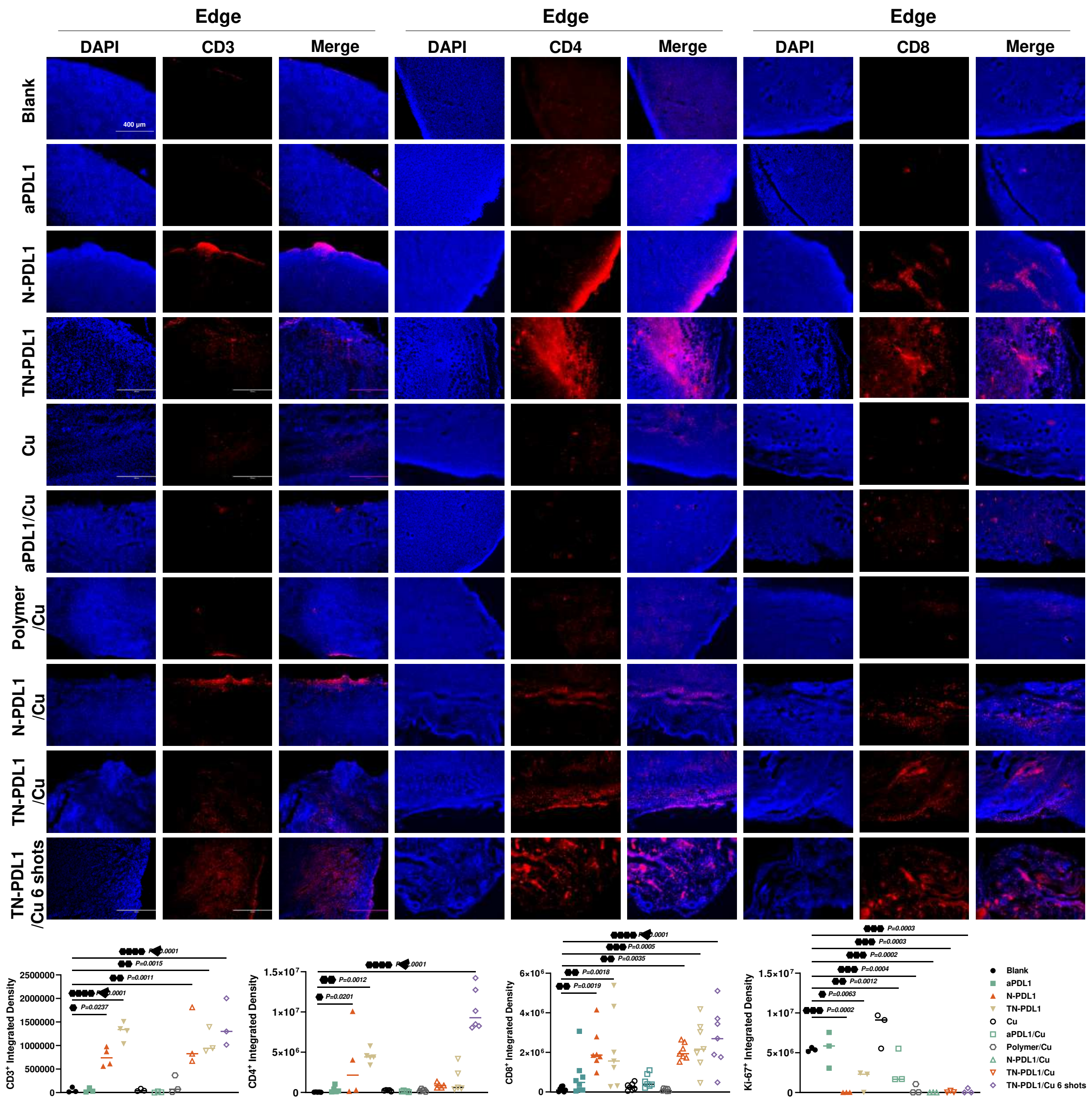

Supplementary Fig. 9. Immunofluorescence images and quantitative analysis of the T cell infiltration on the edge of the tumors. The presence of T cell subpopulations, including $CD3^+$, $CD4^+$, and $CD8^+$ T cells, were detected by immunohistochemistry. The nuclei were stained with Hoechst 33342 (blue). Scale bars = 400 µm. Statistical analysis was performed via ANOVA one-way test, n=3~6, Mean ± SD, *****P < 0.0001*.
